# Supplementary material for: Hybrid Models and Biological Model Reduction with PyDSTool
Source: PLoS Comput Biol. 2012 Aug 9;8(8):e1002628. doi: 10.1371/journal.pcbi.1002628 (PMC3415397; doi:10.1371/journal.pcbi.1002628)
Supplement: Text S4 — Complete source code for the PyDSTool package (version 0.88.120504). Includes API documentation and help files linking to web pages. This file is identical to the current public release on Sourceforge.net. (ZIP) [file pcbi.1002628.s004.zip › PyDSTool/html/PyDSTool.ModelConstructor'.ModelConstructor-class.html]

xml version="1.0" encoding="ascii"?


PyDSTool.ModelConstructor'.ModelConstructor


| Home | Trees | Indices | Help | PyDSTool |
| --- | --- | --- | --- | --- |

Package PyDSTool ::
Module ModelConstructor' ::
Class ModelConstructor

[hide private]

[frames] | no frames]

# Class ModelConstructor

source code

```
object --+
         |
        ModelConstructor
```

---


|  |  |
| --- | --- |
| Instance Methods | [hide private] |

|  |  |
| --- | --- |
| \_\_init\_\_(self, name, userevents=None, userfns=None, unravelInfo=True, inputs=None, checklevel=2, activateAllBounds=False, generatorspecs=None, indepvar=`(``'``t``'``,` `[`-inf`,` inf`]``)`, parvalues=None, icvalues=None, tdata=None, reuseTerms=None, withJac=None, withJacP=None, featureDicts=None, abseps=None, eventtol=None, eventPars=None, withStdEvts=None, stdEvtArgs=None)  Notes for initialization arguments. | source code |

|  |  |
| --- | --- |
| \_\_repr\_\_(self)  repr(x) | source code |

|  |  |
| --- | --- |
| \_genFromMSpec(self, geninfodesc) | source code |

|  |  |
| --- | --- |
| \_genFromStrings(self, geninfodesc) | source code |

|  |  |
| --- | --- |
| activateBounds(self, varname=None, which\_bounds=`'``all``'`)  which\_bounds argument is either 'all', 'lo', 'hi', or a pair ('lo', 'hi'). | source code |

|  |  |
| --- | --- |
| addEvents(self, hostGen, evTarg, eventPars=None) | source code |

|  |  |
| --- | --- |
| addFeatures(self, hostGen, featDict)  Update with feature -> Bool mapping dictionaries for a host generator. | source code |

|  |  |
| --- | --- |
| addFunctions(self, hostGen, fnTarg) | source code |

|  |  |
| --- | --- |
| addModelInfo(self, genSpec, genTarg, genAlgPars=`{``}`, unravelInfo=`{``}`, genOpts=`{``}`)  genSpec can either be a complete ModelSpec description or a string-based dictionary of definitions. | source code |

|  |  |
| --- | --- |
| createGenerators(self)  Create the generators from the source specs, either in the form of dicts or args objects, or as a GDescriptor. | source code |

|  |  |
| --- | --- |
| createJac(self) | source code |

|  |  |
| --- | --- |
| createJacP(self) | source code |

|  |  |
| --- | --- |
| createStdEvts(self) | source code |

|  |  |
| --- | --- |
| getModel(self)  Build and return (hybrid) model made up of declared Generators and the mappings between events used to change vector fields in a hybrid system. | source code |

|  |  |
| --- | --- |
| mapEvent(self, hostGen, eventname, target, eventmapping=None)  eventmapping may be a dictionary or an EvMapping product. | source code |

|  |  |
| --- | --- |
| preprocessFlatten(self) | source code |

|  |  |
| --- | --- |
| preprocessReuseTerms(self) | source code |

|  |  |
| --- | --- |
| setInternalVars(self, arg) | source code |

|  |  |
| --- | --- |
| setReuseTerms(self, rdict) | source code |

**Inherited from `object`**:
`__delattr__`,
`__getattribute__`,
`__hash__`,
`__new__`,
`__reduce__`,
`__reduce_ex__`,
`__setattr__`,
`__str__`


|  |  |
| --- | --- |
| Properties | [hide private] |

**Inherited from `object`**:
`__class__`


|  |  |
| --- | --- |
| Method Details | [hide private] |

|  |  |
| --- | --- |
| \_\_init\_\_(self, name, userevents=None, userfns=None, unravelInfo=True, inputs=None, checklevel=2, activateAllBounds=False, generatorspecs=None, indepvar=`(``'``t``'``,` `[`-inf`,` inf`]``)`, parvalues=None, icvalues=None, tdata=None, reuseTerms=None, withJac=None, withJacP=None, featureDicts=None, abseps=None, eventtol=None, eventPars=None, withStdEvts=None, stdEvtArgs=None)  *(Constructor)* | source code |

```
Notes for initialization arguments.

name : string name of this ModelConstructor

** The following are applied to all Generators

activateAllBounds : Boolean
checklevel : integer
indepvar : pair of (independent var name, pair giving domain interval)

** The following are dictionaries keyed by Generator name, with values:

generatorspecs : ModelSpecs
userevents : list of Event objects
userfns : dictionary of named user functions specs
inputs : dictionary of Variable objects
algparams : dictionary of algorithmic parameters for Generator
parvalues : dictionary of parameter values
icvalues : dictionary of initial condition values
reuseterms : dictionary of reused terms in specifications
eventPars : list of parameter names associated solely with events
withStdEvts : Boolean for making standard events (bounds & turning points)
stdEvtArgs : arguments for the standard events
featureDicts : dictionary of Features for making each Model Interface
withJac : Boolean for making Jacobian
withJacP : Boolean for making Jacobian with respect to parameters

RETURNS: Nothing, but getModel method returns an instantiated Model
  object when the specifications are complete and consistent.
```

Overrides: object.\_\_init\_\_

|  |  |
| --- | --- |
| \_\_repr\_\_(self)  *(Representation operator)* | source code |

repr(x)

Overrides: object.\_\_repr\_\_: *(inherited documentation)*

|  |  |
| --- | --- |
| activateBounds(self, varname=None, which\_bounds=`'``all``'`) | source code |

which\_bounds argument is either 'all', 'lo', 'hi', or a pair ('lo',
'hi'). Calling with no arguments defaults to activating all bounds.

|  |  |
| --- | --- |
| createGenerators(self) | source code |

Create the generators from the source specs, either in the form of
dicts or args objects, or as a GDescriptor.

Still some teething trouble getting expected types neat and tidy.

|  |  |
| --- | --- |
| mapEvent(self, hostGen, eventname, target, eventmapping=None) | source code |

eventmapping may be a dictionary or an EvMapping product. You must
have declared all generators before calling this function!

  


| Home | Trees | Indices | Help | PyDSTool |
| --- | --- | --- | --- | --- |

|  |  |
| --- | --- |
| Generated by Epydoc 3.0.1 on Fri May 4 15:24:07 2012 | http://epydoc.sourceforge.net |
